# Electrodeposition of tin nanowires from a dichloromethane based electrolyte†

**DOI:** 10.1039/c8ra03183e

**Published:** 2018-07-02

**Authors:** Andrew W. Lodge, Mahboba M. Hasan, Philip N. Bartlett, Richard Beanland, Andrew L. Hector, Reza J. Kashtiban, William Levason, Gillian Reid, Jeremy Sloan, David C. Smith, Wenjian Zhang

**Affiliations:** Chemistry, University of Southampton, Highfield Southampton SO17 1BJ UK A.L.Hector@soton.ac.uk; Department of Physics, University of Warwick Coventry CV4 7AL UK; Physics and Astronomy, University of Southampton, Highfield Southampton SO17 1BJ UK

## Abstract

Tin was electrodeposited from a dichloromethane-based electrolyte at ambient temperature into gold coated anodic alumina membranes with nanoscale pores. The tin nanowires are mainly 〈200〉 aligned, together with some 〈101〉 and 〈301〉 wires. Partial filling of the structure and a distribution of wire lengths was found. Grafting of the pores with hydrophobic surface groups was trialled as a means of modifying the deposition, however, it did not increase the proportion of pores in which wires grew. Under potentiostatic conditions the limited rates of nucleation and diffusion down the 1D pores control the growth of the nanowires.

## Abstract 2

Tin was electrodeposited from a dichloromethane-based electrolyte at ambient temperature into gold coated anodic alumina membranes with nanoscale pores.

## Graphical abstract

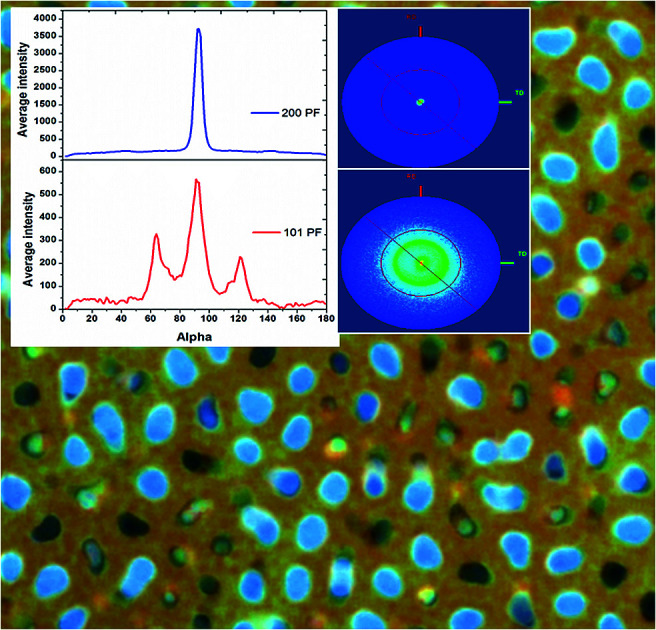

## Introduction

Significant recent investigation has been undertaken into the electrodeposition of nanowires of various metals and dimensions. The unique conductivity behaviour of many materials at the nanowire scale^1^ has also led to investigation towards their use in thermoelectric materials,^2–6^ solar energy,^1,7^ batteries and capacitors,^8,9^ and sensors.^1,3,10–12^

One method to form nanowires is to electrodeposit conducting materials inside the pores of porous anodic aluminium oxide (AAO) membranes. This has previously been employed in the production of nanowires of Ni,^13,14^ Co,^13,14^ Cu,^13,14^ Fe,^13,14^ Pd,^10,15^ Au,^16^ Sb,^17^ Bi,^3^ Ag^5^ and Si,^18,19^ as well as alloys of Pd–Ni,^20^ Pd–Fe,^21^ Pt–Co,^22^ Ni–Ga^23^ and Si–C,^24^ and has the advantage that one end of the wire is electrically contacted by virtue of the electrodeposition method employed. One potential problem with electrodeposition into a porous structure is the narrow pore diameter and associated high aspect ratio, causing restricted diffusion of the metal ions to the bottom of the pore. The surface tension and viscosity of the deposition solution are critical to managing this challenge. Aqueous electrodeposition solutions have a high surface tension, which can prevent the solution from reaching the bottom of a pore. In addition, any gases evolved due to water decomposition result in bubbles that can block access of the reagents to the electrode. Therefore, alternative solvents with lower surface tensions and greater electrochemical stability are being investigated.

Non-aqueous solvents used for the electrodeposition of metals include dimethylsulfoxide,^25^ methanol,^26^ ethanol,^27,28^ acetone,^27^ acetonitrile,^26^ propylene carbonate^26^ and ionic liquids.^29–32^ Supercritical fluids are a good option, as electrolytes can be designed with densities, conductivities and solubilities similar to those observed in liquids, but they have a negligible surface tension, and any substances that would be evolved in the gas phase are instead evolved in the supercritical phase, reducing any electrode-blocking effects or turbulence. This has led to investigation into their use for electrodeposition into porous structures,^4,33–35^ and we have recently reported the growth of tin nanowires from a supercritical CH_2_F_2_-based electrolyte.^35^ Dichloromethane (CH_2_Cl_2_) has low surface tension and is a good solvent for the preparation of electrodeposition baths,^36^ but can be handled without the high pressure systems needed for supercritical solvents. However, there are also disadvantages to CH_2_Cl_2_, including its environmental effects and relatively high volatility.

The penetration of the electrolyte and transport to the bottom of the anodic alumina pores can be modified by changing the chemical properties of the pore wall to increase interactions with the electrolyte solvent. Chemical modification of the surface of anodic alumina pore walls has been investigated by several groups.^37–40^ For example, silane groups have been grafted to the internal pore wall of the membranes to increase the hydrophobic character of the membrane and aid in the transport of hydrophobic molecules.^38,39,41^ Here a number of grafting procedures are tested for their effects on tin deposition from CH_2_Cl_2_-based electrolytes.

Tin is a versatile material which is commonly used in corrosion resistant surface coatings, electrical connectors for integrated circuits and (in alloys) as solder materials. Below 13 °C the stable tin phase changes from the semi-metallic β-tin to semiconducting α-tin.^42^ It is predicted that in nanowires with diameters of less than 3 nm, a bandgap can be induced due to this same change in the stable phase.^43^ Nanowires of tin may have potential uses as infrared detectors due to the small forbidden energy band gap (0.1 eV).^42^ Some single crystal tin nanowires have been observed displaying superconducting behaviour at low temperatures (3.7 K).^44^ Nanowire tin has also been investigated for use in lithium-ion batteries.^9^ Some research has been undertaken into the synthesis of tin in anodic alumina membranes.^9,45,46^ Chen *et al.* used hydraulic pressure to force molten Sn through an AAO membrane to produce nanowires of 15 and 60 nm.^46^ Kim *et al.* electrodeposited wires of 2 μm length with diameters of between 50 and 100 nm in AAO membranes.^9^ Luo *et al.* electrodeposited tin *via* the deposition of copper nanorods in the base of the pore followed by the deposition of tin in the rest of the pore.^45^ This produced aligned tin with diameters of up to 100 nm.^45^ The previous electrodeposition work used aqueous electrolytes, with the exception of our recent supercritical fluid study.^47^ Herein, the utility of CH_2_Cl_2_-based electrolytes is explored.

## Experimental

The precursor compound [N^*n*^Bu_4_][SnCl_3_] was prepared and characterised as previously described.^36^ Anhydrous [N^*n*^Bu_4_]Cl (Fluka, >99.0%) was used as received, dichloromethane (CH_2_Cl_2_, Fisher, ACS reagent grade) was distilled from CaH_2_, tetrahydrofuran (THF, Fisher, ACS reagent grade) was distilled from sodium benzophenone ketyl ether and ethanol (Fisher, absolute) was distilled from a sodium ethoxide solution. Diethylphenylphosphonate (C_6_H_5_P(O)(OCH_2_CH_3_)_2_) was synthesised by reacting phenylphosphonic dichloride (Sigma, 90%) with dry ethanol in an ice bath, then removing the solvent *in vacuo* to remove HCl and drive the equilibrium.^48^ Trimethylchlorosilane ((CH_3_)_3_SiCl, Sigma, >99%) and ethyltrimethoxysilane (C_2_H_5_Si(OCH_3_)_3_, TCI, >97%) were used as purchased. These compounds were stored and handled in a dry nitrogen atmosphere.

Anodic alumina membranes were purchased from Whatman (200 nm pores, 13 mm diameter circles, ∼60 μm thickness) or Synkera Industries (55 or 13 nm pores, 1 × 1 cm squares, ∼50 μm thickness). These were dried at 130 °C for 6 hours under vacuum prior to use and then handled under nitrogen. Evaporation of a 5 nm Cr wetting layer followed by a 200 nm Au film was used to produce gold films on microscope slides for the CV studies and to coat the reverse side of membranes used for wire depositions. In some cases membrane surfaces were grafted before metal coating using C_6_H_5_P(O)(OCH_2_CH_3_)_2_, (CH_3_)_3_SiCl or C_2_H_5_Si(OCH_3_)_3_ under N_2_. The grafting agent (0.5 cm^3^) was added dropwise onto the surface of the membrane. CH_2_Cl_2_ (40 cm^3^, for C_6_H_5_P(O)(OCH_2_CH_3_)_2_) or THF (10 cm^3^, for silanes) was added to the flask and refluxed at 75 °C for 24 hours. The grafting solution was decanted and the membrane was washed with 5 × 10 cm^3^ portions of THF or CH_2_Cl_2_, followed by portions of ethanol before drying at 130 °C *in vacuo* for 6 hours.

Cyclic voltammetry and potential step chronoamperometry were undertaken on a Bio-logic SP-150 potentiostat using EC-Lab software V10.40. Working electrodes were a region of a gold-backed anodic alumina membrane defined by a 4 mm internal diameter O-ring in a custom-built cell (ESI, Fig. S1†). The electrolyte containing 0.01 mol dm^−3^ [N^*n*^Bu_4_][SnCl_3_] and 0.1 mol dm^−3^ [N^*n*^Bu_4_]Cl in CH_2_Cl_2_ was prepared and handled under N_2_. The N_2_-flushed 3-electrode cells were also fitted with a Ag/AgCl reference electrode (0.1 mol dm^−3^ [N^*n*^Bu_4_]Cl in CH_2_Cl_2_) and a platinum gauze counter electrode.

Scanning electron microscopy (SEM) used a Zeiss Gemini SEM 500 operating at 1–10 kV and a Jeol JSM-6500F FEG-SEM operating at 15 keV. Transmission electron microscopy (TEM) specimens were prepared using conventional mechanical polishing followed by ion milling to electron transparency using Ar^+^ at 6 keV. A final low-energy milling step was performed at 500 eV in order to minimize surface damage. The structure and morphology of the samples were analysed using a Jeol 2100 TEM equipped with a LaB_6_ electron source and Jeol ARM200F TEM/scanning TEM (STEM) with a Schottky gun both operating at 200 kV. Annular dark-field (ADF) STEM measurement was performed in the ARM200F, with probe and image aberration CEOS correctors. ADF-STEM images were obtained using a Jeol annular field detector with a probe current of approximately 23 pA, a convergence semi-angle of ∼25 mrad, and an inner angle of 45–50 mrad. An Oxford Instruments X-MaxN 100TLE windowless silicon drift detector (SSD) was used to perform STEM-EDX analysis.

X-ray diffraction (XRD) patterns were collected at a grazing incidence angle of 1° to maximise the signal from the deposit, and in parallel beam *θ*–2*θ* geometry to investigate preferred orientation, using Cu-K_α_ radiation on a Rigaku Smartlab Thin Film diffractometer with a DTex 250 1D detector. Phase matching and lattice parameter refinements used the Rigaku PDXL2 package, with standard patterns from the JCPDS^49^ and ICSD^50^ databases. Pole figure measurements used either in-plane geometry or *θ*–2*θ* geometry with the diffraction vector orientation controlled by a *χ* circle rotation. Water contact angle measurements were made on a Kruss DSA100 using DSA v1.90.0.11 software and using a 1 μL water droplet on the surface.

## Results and discussion

We previously reported tin electrodeposition onto gold disk electrodes^36^ and into anodic alumina membranes^47^ using [N^*n*^Bu_4_][SnCl_3_] in supercritical CH_2_F_2_. In this work, we employ the same deposition reagent and supporting electrolyte salt in liquid CH_2_Cl_2_ at ambient pressure to deposit into membranes.

**Fig. 1:**
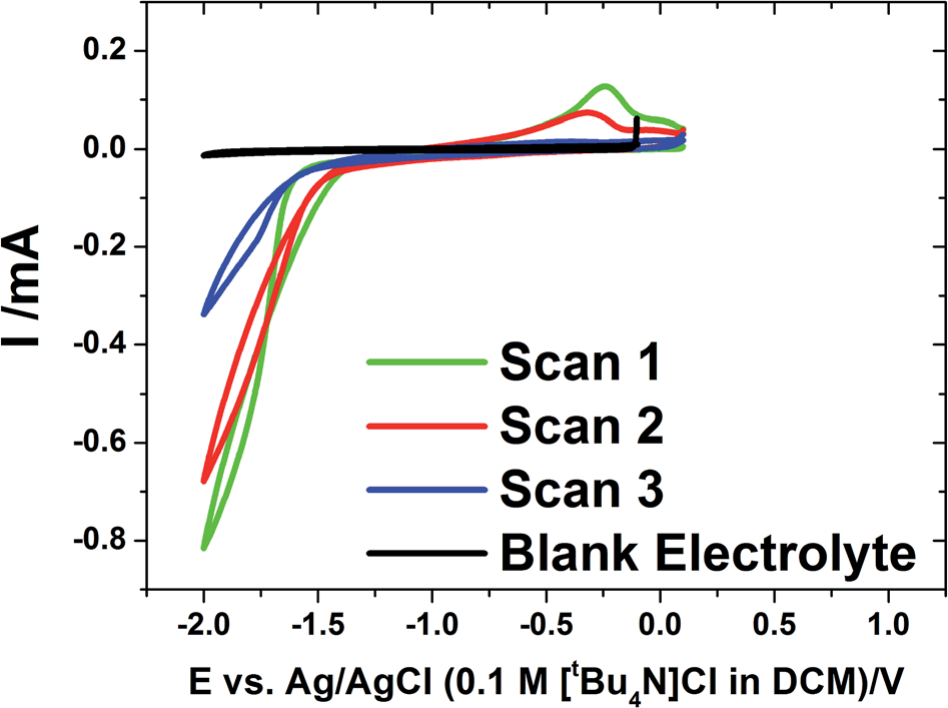
Cyclic voltammograms at 0.05 V s^−1^ for the electrolyte containing 0.01 mol dm^−3^ [N^*n*^Bu_4_][SnCl_3_] and 0.1 mol dm^−3^ [N^*n*^Bu_4_]Cl in CH_2_Cl_2_. The voltammograms were undertaken at ambient temperature (approximately 20 °C). The working electrode was a 1 cm^2^ gold film. The blank electrolyte contained 0.1 mol dm^−3^ [N^*n*^Bu_4_]Cl in CH_2_Cl_2_ but no tin reagent.

Cyclic voltammetry was undertaken on evaporated gold on glass electrodes in a solution of [N^*n*^Bu_4_][SnCl_3_] in CH_2_Cl_2_ with a [N^*n*^Bu_4_]Cl supporting electrolyte at ambient temperature (approximately 20 °C) (Fig. 1). The voltammetry showed a single, large reduction wave feature on the cathodic scan below ∼−1.3 V, followed by a tin stripping peak ∼−0.25 V on the reverse scan. The scans show some IR drop due to low electrolyte conductivity.

**Fig. 2:**
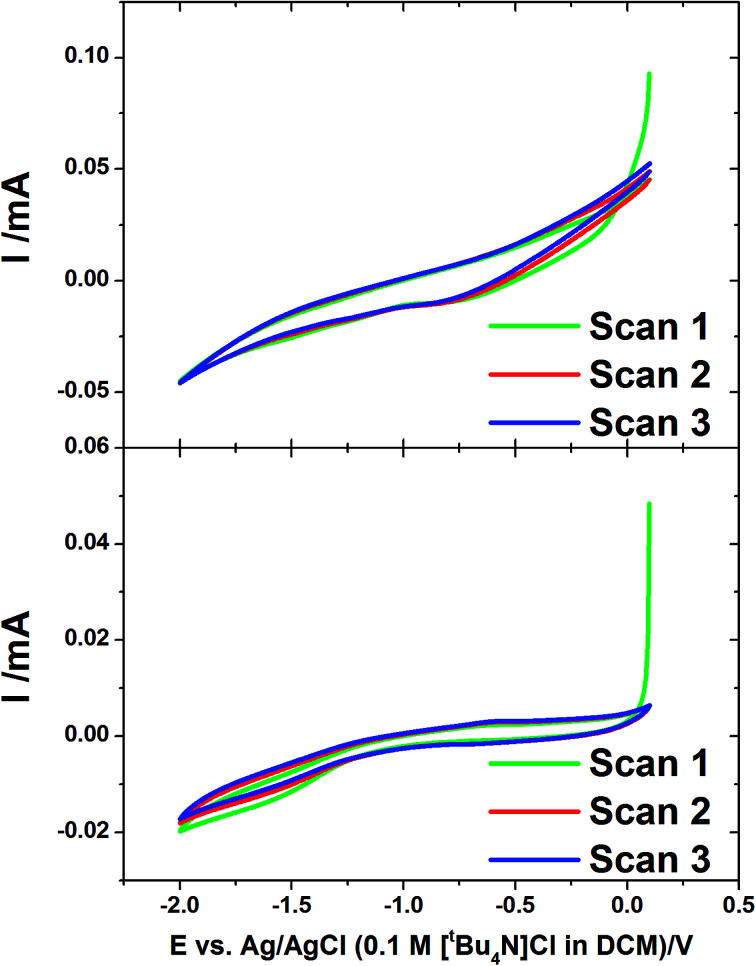
Cyclic voltammograms of AAO membranes with 55 nm (upper) and 13 nm (lower) pore diameter. These were undertaken in electrolyte containing 0.01 mol dm^−3^ [N^*n*^Bu_4_][SnCl_3_] and 0.1 mol dm^−3^ [N^*n*^Bu_4_]Cl in CH_2_Cl_2_ with a scan rate of 50 mV s^−1^.

Cyclic voltammetry was also undertaken at the gold-coated AAO membranes in the tin deposition solution at 50 mV s^−1^ (Fig. 2). The voltammograms show a much greater IR drop than was observed on flat gold, related to a slow rate of diffusion in the pore. The voltammograms both show a reduction wave which starts to occur at −1.0 V corresponding to the onset of the deposition of tin in the membranes. This is most obvious on the CV in a 13 nm pore diameter membrane which shows a significant curve occurring at around −1.5 V. The reverse scans show no significant features indicating no significant stripping of tin in the pores.

### Tin electrodeposition into AAO membranes

**Fig. 3:**
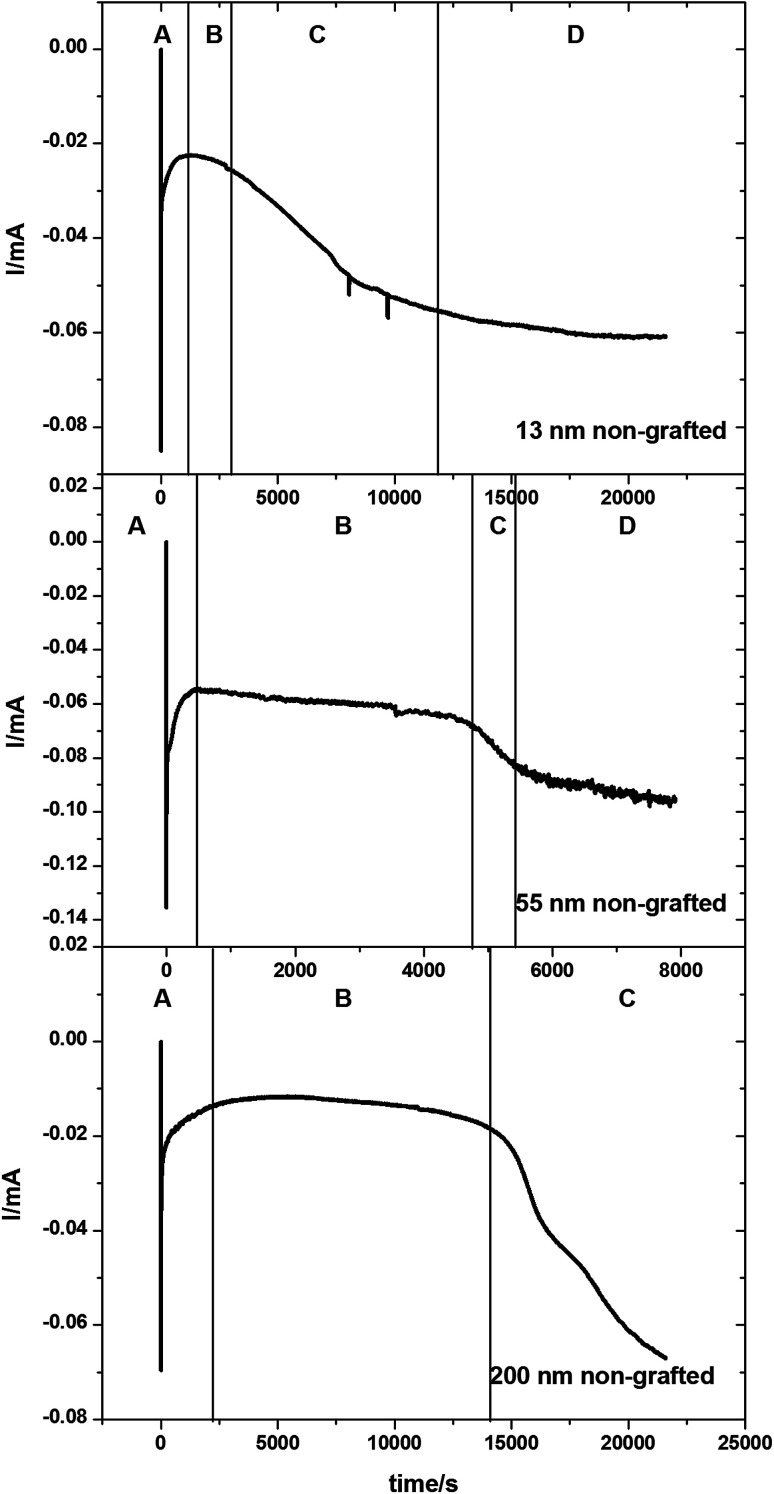
Potential step chronoamperograms for Sn electrodeposited into a gold-coated, 13 nm, 55 nm and 200 nm pore diameter anodic alumina membranes at −1.5 V. The chronoamperograms were collected with an electrolyte consisting of 0.01 mol dm^−3^ of [N^*n*^Bu_4_][SnCl_3_] with 0.01 mol dm^−3^ [N^*n*^Bu_4_]Cl in CH_2_Cl_2_. (A) Initial Sn nucleation and deposition; (B) diffusion-limited Sn deposition inside the pores; (C) overgrowth of Sn on the surface of the membrane once the growth reaches the open surface of the membrane; (D) deposition of Sn on the surface of the membrane once all the pores are filled.

Tin deposition was carried out on gold coated AAO membranes with pore diameters of 13, 55 and 200 nm. The electrodeposition was undertaken using the same solution as described above. All depositions were undertaken at ambient temperature. A deposition potential of −1.5 V *vs.* Ag/AgCl was applied for a period of up to 6 hours, or until the amperometry showed a large increase or plateau in the current, indicating deposition exclusively on the surface of the membranes. Sample chronoamperograms are shown in Fig. 3. These chronoamperograms all showed several similar features. Initially there is a large increase in the current (Region A) which rapidly decreases to reach a steady state current (Region B). After a certain amount of time at the steady state the current increases (Region C) before plateauing off again (Region D).

The initial nucleation of tin at the bottom of the pores causes the current to decrease (Region A) until it reaches a steady-state diffusion-limited current at the start of the second region (Region B). The deposition of the tin inside the pores as nanowires reduces the path length in the diffusion-limited growth and hence the current gradually increases. After several hours of electrodeposition, some pores are completely filled and the current increases (Region C) as the tin breaks through onto the surface of the membrane and diffusion occurs more readily to the surface growth sites.^52–54^ An increase in convection noise is also observed in this region of the chronoamperogram as tin is then deposited preferentially onto the surface of the membrane (Region D).

In Fig. 3 the amount of time spent in Region B varies with pore sizes, with the smaller 13 nm diameter pore membranes taking approximately 3000 seconds to reach Region C, the 55 nm pore diameter membranes taking approximately 5000 seconds to reach Region C and the largest 200 nm pore diameter membranes taking over 15 000 seconds before the breakthrough of tin onto the surface of the membrane. This is related to the increasing volume of the pore to be filled before breakthrough. It is also of note that the 200 nm pore diameter membranes do not reach region D within the experimental timescale.

**Fig. 4:**
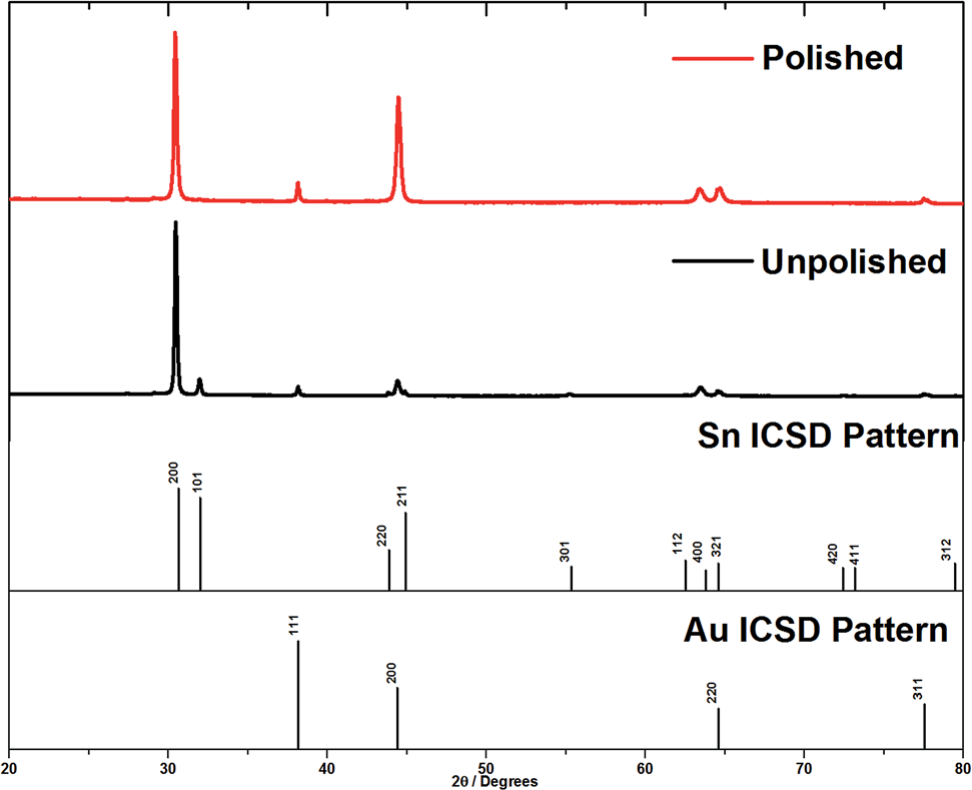
*θ*–2*θ* diffraction patterns of tin deposits grown on gold coated AAO membranes with a nominal pore diameter of 55 nm. This shows the effect of removing the surface layer of tin.

X-ray diffraction patterns were collected in *θ*–2*θ* mode to penetrate into the surface of the membrane, and then again after polishing the surface to remove the over-growth. Tetragonal β-tin was detected in all membranes used for deposition. Prior to polishing, all of the expected Sn peaks were observed (Fig. 4), but a reasonably strong 〈200〉 preferred orientation was observed. In the *θ*–2*θ* mode the diffraction vector is along the axis of the nanowires, so this suggested a mixture of 〈200〉 aligned tin wires and polycrystalline tin on the surface of the AAO. Note that the Au 111, 200 and 220 peaks are also visible in the membrane XRD pattern, so the X-ray beam is clearly penetrating right through the membrane. After removing the surface deposits by polishing, the Sn 101 peak was fully suppressed and only the 200 and 400 peaks of the tin pattern were observed. This confirms that the main orientation of the wires inside the pores of the AAO was 〈200〉. Polishing the membrane also causes an increase in the Au 200 peak intensity. This large Au 200 peak is also seen on XRD of unpolished AAO membranes suggesting that the evaporation process causes it to be aligned.

**Fig. 5:**
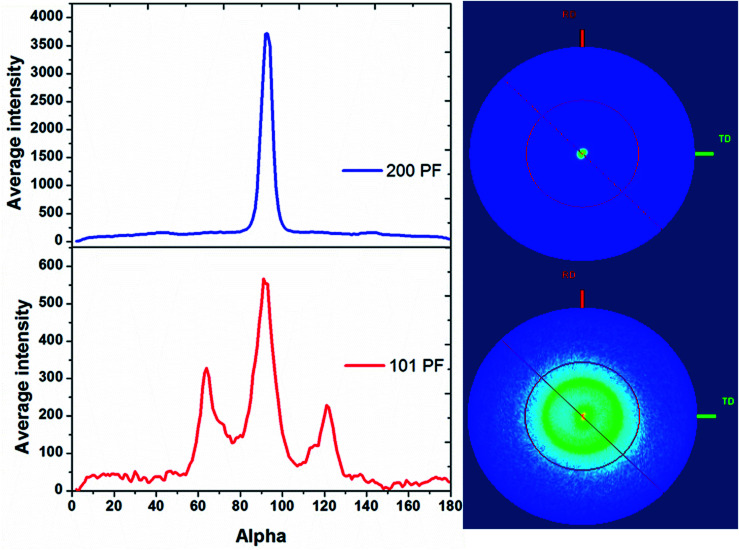
〈200〉 (top) and 〈101〉 (bottom) pole figure measurements on Sn deposited into a 13 nm pore diameter AAO membrane, with the cross-sectional integrals on the left and the 2D projections on the right. The Sn deposition was undertaken at −1.5 V *vs.* Ag/AgCl, in 0.01 mol dm^−3^ [N^*n*^Bu_4_][SnCl_3_] and 0.01 mol dm^−3^ [N^*n*^Bu_4_]Cl in CH_2_Cl_2_ at room temperature, and the sample was polished to remove surface overgrowth before these measurements.

Pole figure measurements on the tin in the 13 nm pore diameter AAO membrane are shown in Fig. 5. The Sn 200 pole figure shows an intense central peak indicative of strong Sn 〈200〉 orientation in the membrane, as expected from the *θ*–2*θ* scans. The Sn 101 pole figure also shows a central peak at 90° surrounded by a smaller ring at approximately 31° from the centre of the pole figure. This indicates the presence of a significant fraction of Sn wires with 〈101〉 orientation and a small number with 〈301〉 orientation. The intensities of the integrals show that the Sn 〈200〉 orientation is 6–7 times more intense than the 〈101〉. Pole figures on tin in AAO membranes with pore diameters of 55 and 200 nm are shown in ESI, Fig. S2 and S3.† These also show a strong Sn 〈200〉 orientation in the 55 and 200 nm pores with a weaker Sn 〈101〉 orientation. As the pore diameter increases, the degree of Sn 〈200〉 orientation increases and the degree of Sn 〈101〉 orientation decreases. This is much more apparent in the pole figure measurements than the *θ*–2*θ* scans due to their greater sensitivity to the orientation of the wires.

**Fig. 6:**
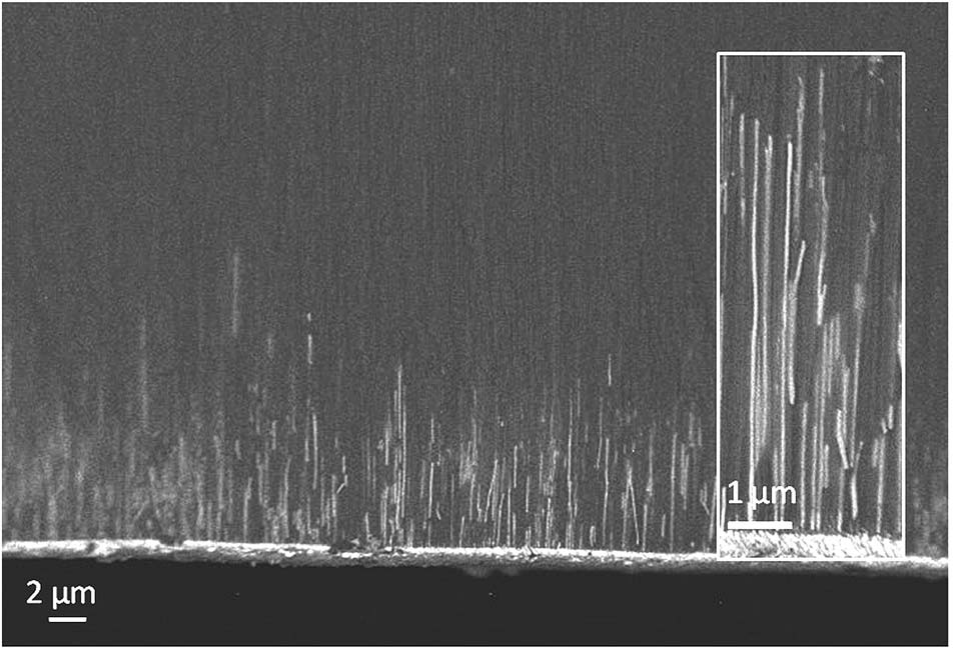
A side-on composition sensitive EsB-SEM image of a cross-section of a 55 nm AAO membrane after electrodeposition of tin until a surface coating had been deposited, showing tin nanowires near the bottom of the pores. The inset shows the wires at a higher magnification.

SEM images of the edge of fractured membranes after tin deposition (Fig. 6) showed that a dense bed of nanowires grew close to the metal plated side of the membrane, with a growth front at a typical distance of 5 μm, around 1/10 of the membrane thickness. Occasional wires are observed higher up in the membrane, but at much lower density. These cannot be followed along their entire length, but that is unsurprising as the fracture surface will not necessarily follow the entire length of the pore. TEM images were initially collected by ion beam milling of the membranes from both sides for similar time periods until thin enough to image. The side with the wires and the gold coating would be expected to etch more slowly, and indeed we did observe wires up to approximately 10 μm long in these samples, both at 13 and 55 nm pore size (ESI, Fig. S4 and S5†). Only around 50% or less of the pores were filled at this height in both membrane pore sizes.

**Fig. 7:**
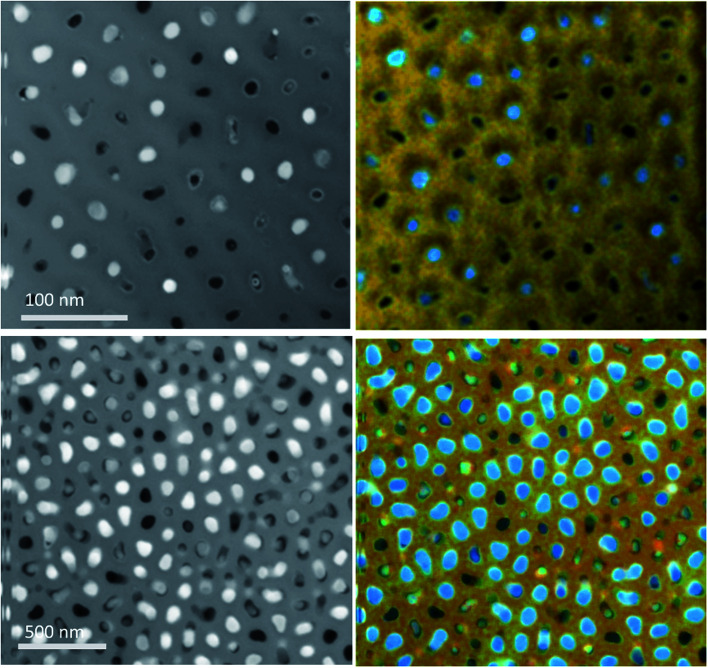
ADF-STEM images (left) and composite STEM-EDX images (right) of a 13 nm (top) and a 55 nm (bottom) pore diameter AAO membrane after electrodeposition of tin until a surface coating had been deposited. In the EDX images Au = Yellow, O = Red, Sn = Blue and Al = Green.

The degree of filling close to the bottom of the pores was tested by etching samples from the gold coated side only just enough to break through the gold, then etching from the uncoated side until thin enough to image (Fig. 7). The TEM images of these samples also show that not all pores were filled, but the EDX data indicate that a large fraction of the pores, more than would be obvious from the images, do contain tin.

Fig. 7 also shows several different morphologies for the tin deposited in the membrane. The most common morphology is a nanowire completely filling the pore. Another observed morphology is a wire filling the centre of the pore but not touching the wall of the pore, likely to be the tip of the wire growing up the pore. The third morphology observed is of tin deposited on the wall of the pore but not in the centre, leaving a nanotube. This could occur due to the internal chemistry or structure of the pore wall, as AAO membranes can have differences in the pore shape and diameter even within the same membrane. However, previous groups have also reported that the antidots on AAO membranes can lead to the deposition of nanotubes instead of nanowires,^51^ indicating that gold on the bottom of the pore may affect the deposition of copper in the pore.

A significant proportion of the pores in both 13 and 55 nm pore diameter AAO membranes contained tin nanowires after electrodeposition from our CH_2_Cl_2_-based electrolyte. There are morphology variations in the deposits, and also differences in the lengths of the nanowires within the membrane. These differences could be attributed to a number of factors: (1) the morphology may be linked to the pore wall chemistry; (2) poor electrolyte penetration in some pores could result in some empty pores; (3) differences in the initial nucleation of the tin at the base of the pores could produce different wire lengths and some empty pores; (4) variable growth rates related to diffusion, which could also be related to pore wall chemistry, could explain the different wire lengths.

To test the electrolyte solvent penetration aspect, pore surfaces were grafted with hydrophobic groups. A large increase in the membrane hydrophobicity was found with various grafting agents (ESI, Fig. S6†) but the current transients during deposition were virtually identical to those from the ungrafted membranes (ESI, Fig. S7†). All membranes showed the presence of tin in the XRD patterns (ESI, Fig. S8 and S9†), and SEM and TEM images (ESI Fig. S10 and S11†) showed that the nanowires deposited in the grafted membranes are much longer and penetrate through more of the membrane, up to approximately 20 μm in length, than the wires observed in the ungrafted membranes. However, the TEM images showed no increase in the proportion of pores filled with tin. This suggests that the grafting enhances the diffusion of the tin ions through the pores, increasing the length of the nanowires. However the grafting does not increase the proportion of the pores that contain nanowires. This indicates that grafting does not affect the extent of nucleation in the pores, and that solvent penetration was not a problem in the ungrafted membranes.

**Fig. 8:**
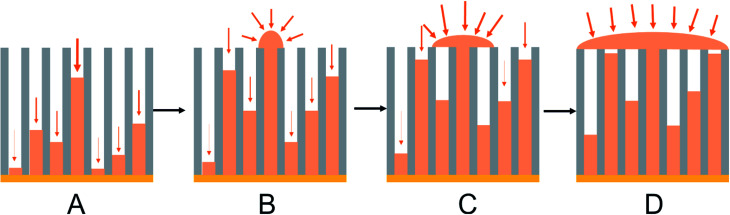
A schematic showing preferential deposition of tin onto the surface of the membrane once a wire reaches the surface adapted from Proenca *et al.*:^51^ (A) initial Sn nucleation and deposition; (B) diffusion-limited Sn deposition inside the pores; (C) overgrowth of Sn on the surface of the membrane once the growth reaches the open surface of the membrane; (D) deposition of Sn on the surface of the membrane once some pores are filled.

The typical anodic alumina growth model described above and in the literature^52–54^ can be modified to take into account the observed growth in ungrafted or grafted tin nanowire samples (Fig. 8). It is clear that in our potentiostatic deposition tin nucleation does not occur in every pore. Nucleation is a stochastic process and will occur at different times in each pore. Each wire will grow from the time when it nucleates, so a distribution of wire lengths will be present. Diffusion will be fastest in the pores where the wire is closest to the surface, so these wires will grow fastest. Once a wire reaches the surface the diffusion pathways to it switch from 1-dimensional to hemispherical and the growth rates become even faster. New nuclei also form on the surface of the emerged wire giving polycrystalline tin alignment. As the growth coats the surface it blocks access to neighbouring pores, with the result that wire growth in the pores is arrested. Overall the result is that only a small number of wires reach the surface, but an overgrown layer of tin can still coat the surface. Nucleation pulses could increase the proportion of pores containing wires, and a careful use of pulsed growth, to limit the effect of the 1-dimensional diffusion down the pores, could be used to achieve a more even growth front. However, we also recently reported electrodeposition of tin into the same 55 nm and 13 nm anodic alumina membranes with a supercritical CH_2_F_2_ solution of the same reagents as used herein.^47^ There a large proportion of the pores were filled with tin, presumably because at the high temperature required to achieve supercritical conditions the tin nucleation rate is faster, and the tin complex has a higher diffusion constant in the supercritical CH_2_F_2_.^4^ Nonetheless, this work shows that electrolytes do not have to be supercritical to penetrate small pores, a high proportion of the pores even in 13 nm pore size anodic alumina membranes did contain tin and it is the differential growth rates in the high aspect ratio pores that result in only small numbers of wires growing to the membrane surface.

## Conclusions

Tin was electrodeposited from a dichloromethane-based electrolyte. X-ray diffraction patterns showed the presence of polycrystalline tetragonal β-tin. Deposition into gold-coated anodic alumina membranes with 13 and 55 nm pore diameters showed strong 〈200〉 alignment, with randomly oriented tin continuing to nucleate onto the surface of the membrane once wires grew through the pores. Under the potentiostatic conditions used, nucleation did not occur in every pore and most wires grew only a fraction of the way through the membrane before the formation of a film on the membrane surface. Grafting the pore surfaces with hydrophobic groups increased the length of the wires deposited, but did not improve the proportion of filled pores, suggesting that solvent penetration was not the main barrier to complete filling of the individual pores but that nucleation and diffusion determine the proportion of pores filled with tin.

## Conflicts of interest

There are no conflicts to declare.

## Supplementary Material

RA-008-C8RA03183E-s001
