# Supplementary material for: Electrodeposition of tin nanowires from a dichloromethane based electrolyte
Source: RSC Adv. 2018 Jul 2;8(42):24013–20. doi: 10.1039/c8ra03183e (PMC9081706; doi:10.1039/c8ra03183e)
Supplement: RA-008-C8RA03183E-s001 [file RA-008-C8RA03183E-s001.pdf]

Electronic Supplementary Material (ESI) for RSC Advances.
This journal is © The Royal Society of Chemistry 2018

Electronic Supplementary Information: Electrodeposition of tin nanowires from a dichloromethane based electrolyte

Andrew W. Lodge,[a] Mahboba M. Hasan,[a] Philip N. Bartlett,[a] Richard Beanland,[b] Andrew L. Hector,[a*] Reza J. Kashtiban,[b] William Levason,[a] Gillian Reid,[a] Jeremy Sloan,[b] David C. Smith[c] and Wenjian Zhang[a]

[a] Chemistry, University of Southampton, Highfield, Southampton, SO17 1BJ, UK

[b] Department of Physics, University of Warwick, Coventry, CV4 7AL, UK

[c] Physics and Astronomy, University of Southampton, Highfield, Southampton, SO17 1BJ, UK

*A.L.Hector@soton.ac.uk

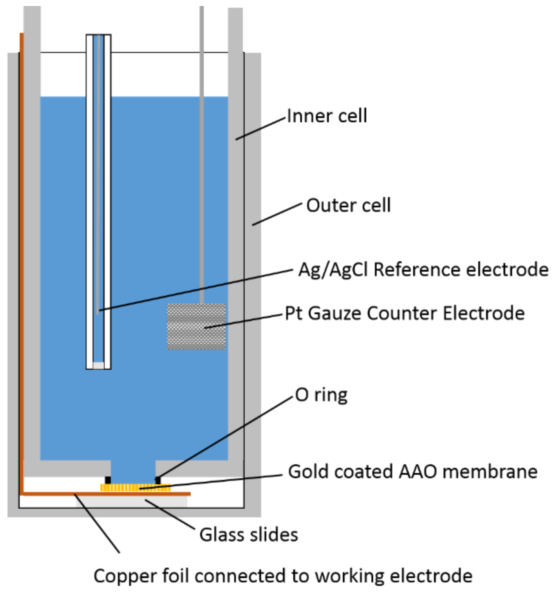

Fig. S1 A schematic showing the design for the cell used to undertake tin electrodeposition into the gold coated AAO membranes.

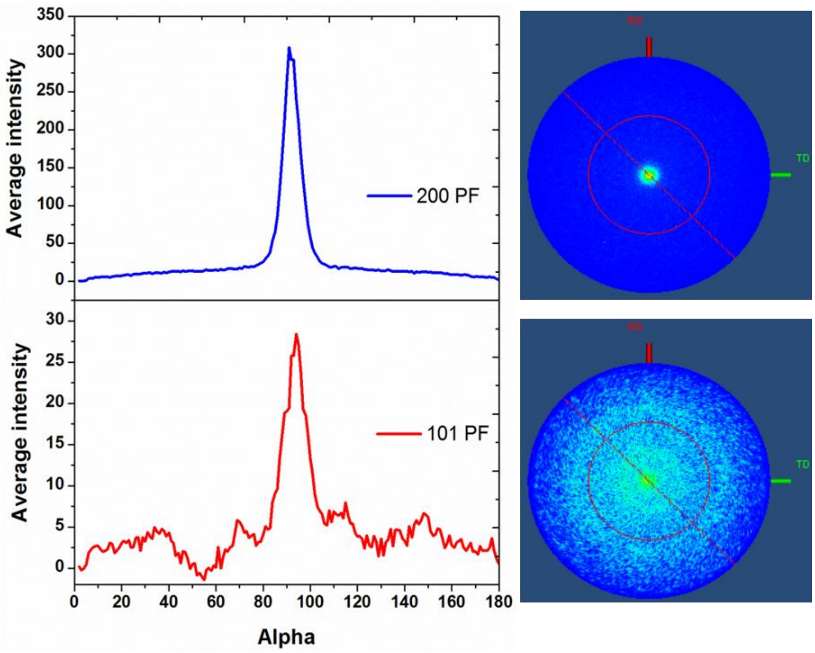

Fig. S2 <200> and <101> pole figure measurements for Sn deposited in an ungrafted AAO membrane with 55 nm diameter pores.

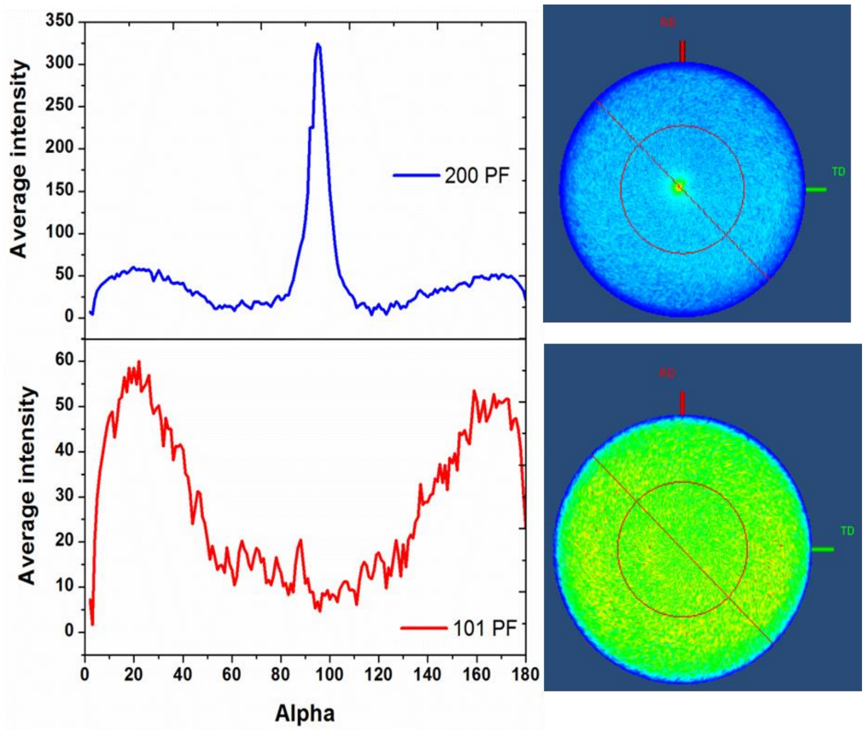

Fig. S3 <200> and <101> pole figure measurements for Sn deposited in an ungrafted AAO membrane with 200 nm diameter pores.

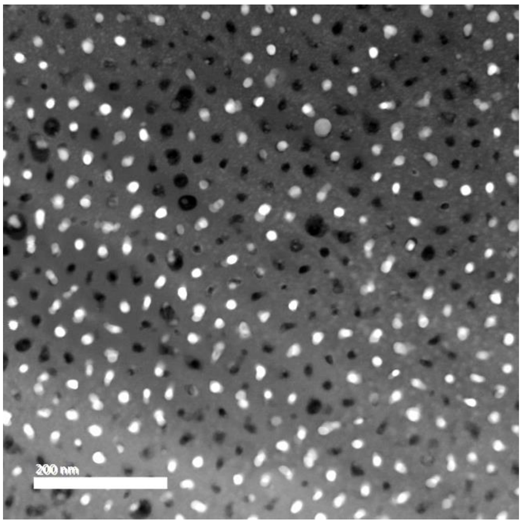

Fig. S4 A STEM-ADF image of a 13 nm pore diameter AAO membrane after Sn electrodeposition showing brighter electrodeposited tin inside the pores. This membrane was sectioned by etching for equal time periods from both sides so the thinned is relatively high up the bed of nanowires.

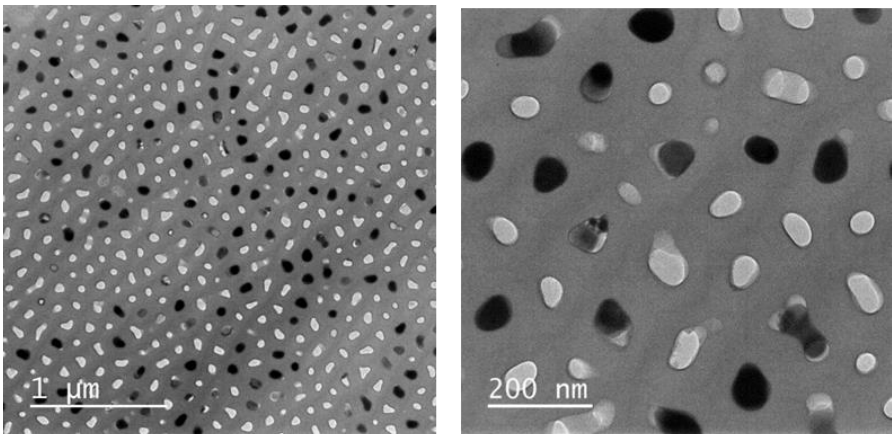

Fig. S5 STEM-BF images of a 55 nm pore diameter AAO membrane after Sn electrodeposition showing darker electrodeposited heavy tin inside the pores. This membrane was sectioned by etching for equal time periods from both sides so the thinned section is relatively high up the bed of nanowires.

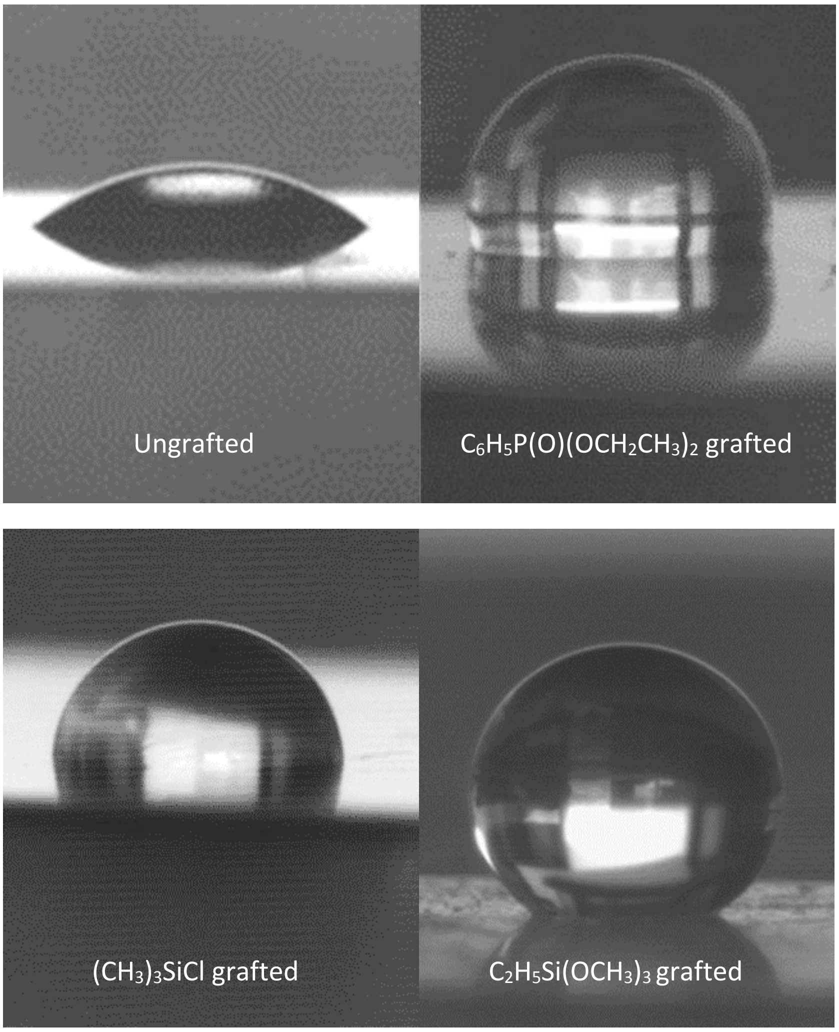

Fig. S6 Contact angle measurements using H_2O on AAO membranes with 55 nm pores both ungrafted and grafted with various functional groups as shown. This shows an increase in the contact angle for water upon grafting the membrane with silane or phosphonate groups.

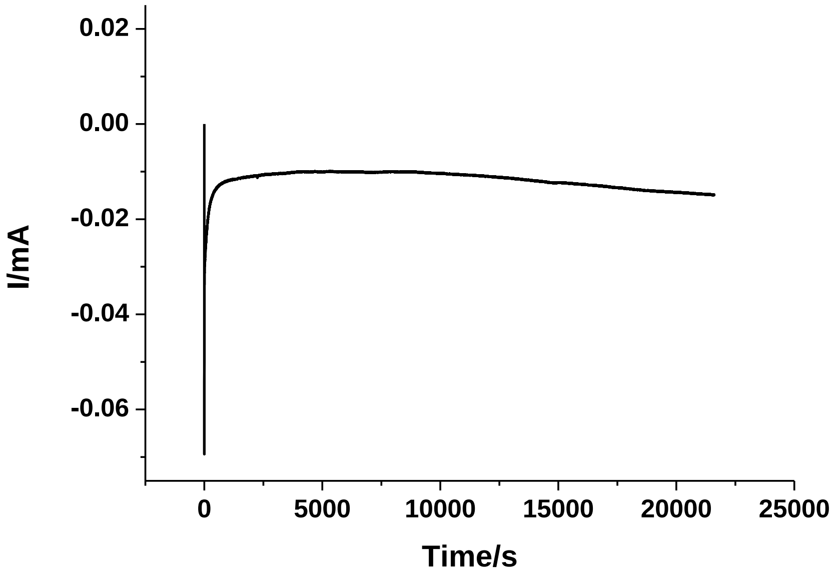

Fig. S7 A potential step chronoamperogram for Sn electrodeposited on a gold coated anodic alumina membrane with 55 nm diameter pores grafted with $C_6H_5P(O)(OCH_2CH_3)_2$. The deposition was at −1.5 V at room temperature. The chronoamperogram was collected in 0.01 mol dm^{-3} [N^{n}Bu$_4$][SnCl$_3$] and 0.1 mol dm^{-3} [N^{n}Bu$_4$]Cl in CH_2Cl_2.

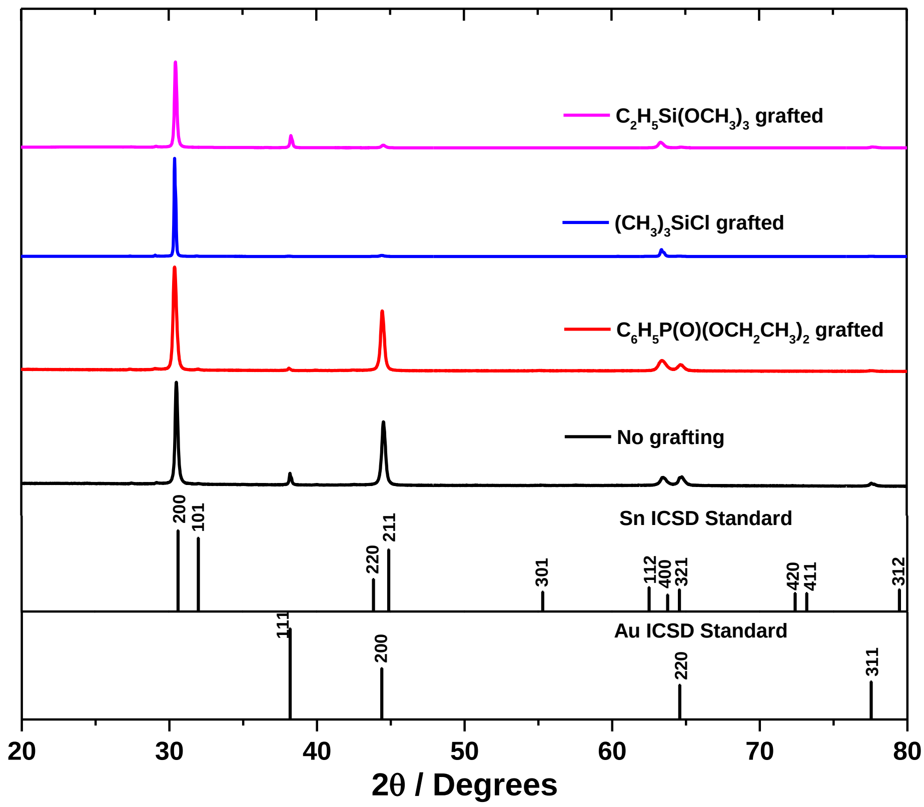

Fig. S8 Symmetric XRD patterns on Sn electrodeposited in 13 nm diameter pore AAO membranes grafted with various functional groups. The Sn deposition was undertaken at −1.5 V *vs*. Ag/AgCl, in 10 mmol dm^{-3} of $[N^nBu_4][SnCl_3]$ and 10 mmol dm^{-3} $[N^nBu_4]Cl$ in CH_2Cl_2 at room temperature. Samples were polished prior to XRD measurement. The ungrafted 13 nm pore diameter membrane shows strong Sn 200 and 400 peaks, as well as the presence of a strong Au 100 peak. This indicates the presence of aligned tin in the pores of the ungrafted membrane. The membranes grafted with $C_6H_5P(O)(OCH_2CH_3)_2$, $(CH_3)_3SiCl$ and $C_2H_5Si(OCH_3)_3$ also show the presence of the Sn <200> phase in the pores of the membrane, indicating the presence of aligned nanowires in the membrane. Comparison of the Sn 200 peaks with the Au 111 and 200 peaks indicates that there may be more tin deposited in the $C_2H_5Si(OCH_3)_3$ and $(CH_3)_3SiCl$ grafted membranes than the ungrafted and $C_6H_5P(O)(OCH_2CH_3)_2$ grafted membranes. This may be due to the increased hydrophobic character of the silane grafted membranes increasing the transport of ions within the CH_2Cl_2 electrolyte.

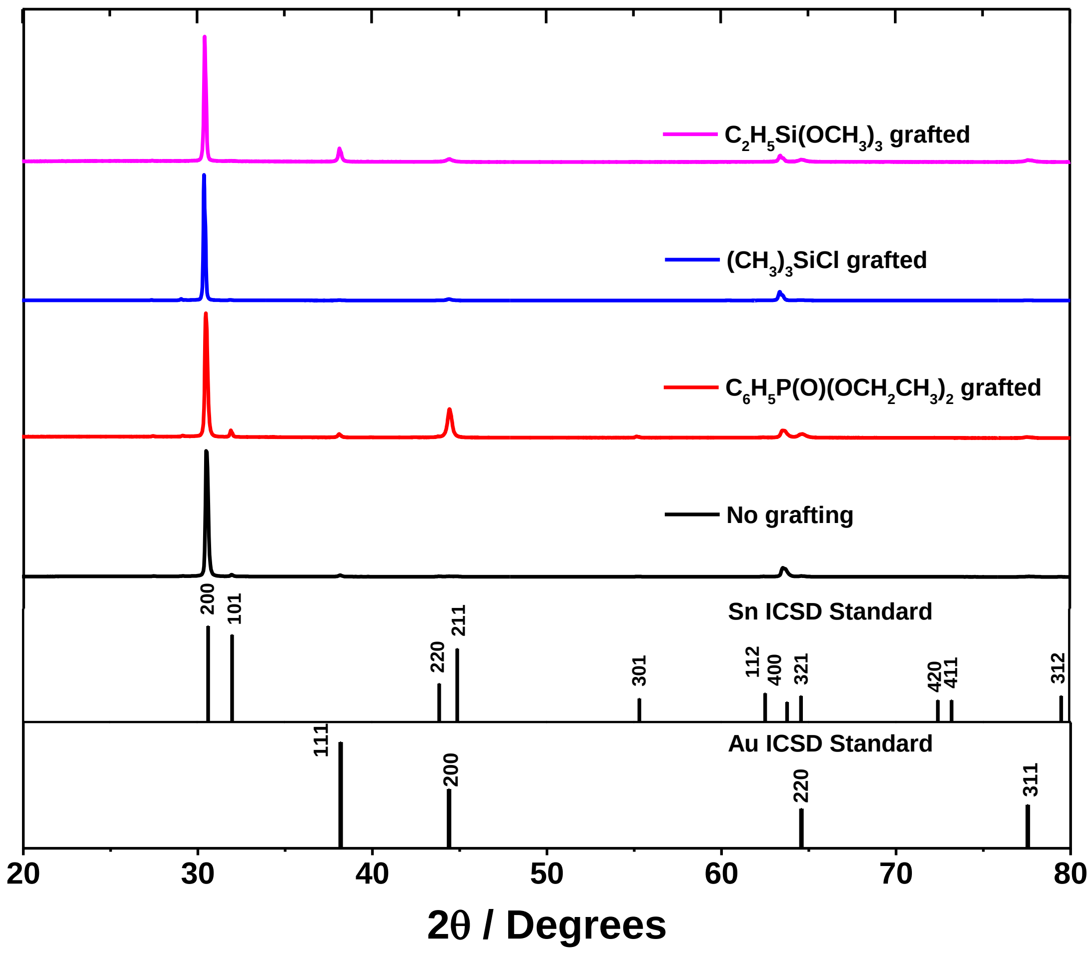

Fig. S9 Symmetric XRD patterns on Sn electrodeposited in 55 nm diameter pore AAO membranes grafted with various functional groups. The Sn deposition was undertaken at −1.5 V *vs*. Ag/AgCl, in 10 mmol dm^{-3} of [N^{n}Bu$_4$][SnCl$_3$] and 10 mmol dm^{-3} [N^{n}Bu$_4$]Cl in CH_2Cl_2 at ambient temperature. Deposition time was varied for each deposition experiment. These patterns show the presence of aligned 200 tin in all of the membranes. Comparison with the Au 111 and 200 peaks indicates that the amount of aligned tin is greatest in the $(CH_3)_3SiCl$ grafted membrane, followed by the $C_2H_5Si(OCH_3)_3$ grafted membrane.

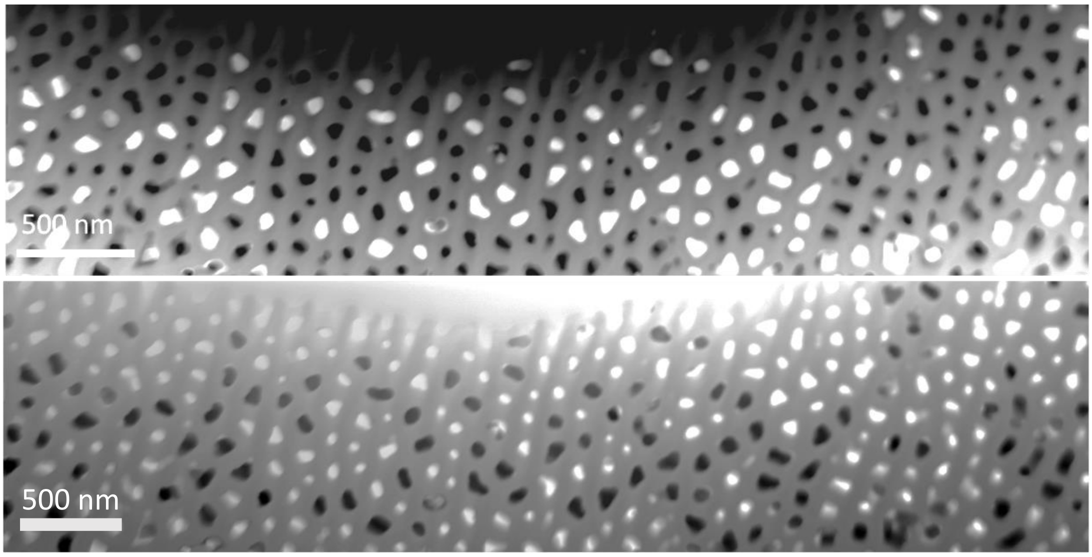

Fig. S10 STEM-ADF (top) and BF (bottom) images showing an anodic alumina membrane with 55 nm diameter pores that have been grafted with $C_2H_5Si(OCH_3)_3$. The heavier tin appears as brighter spots (STEM-ADF) and darker spots (STEM-BF) compared to the membrane walls and empty pores.

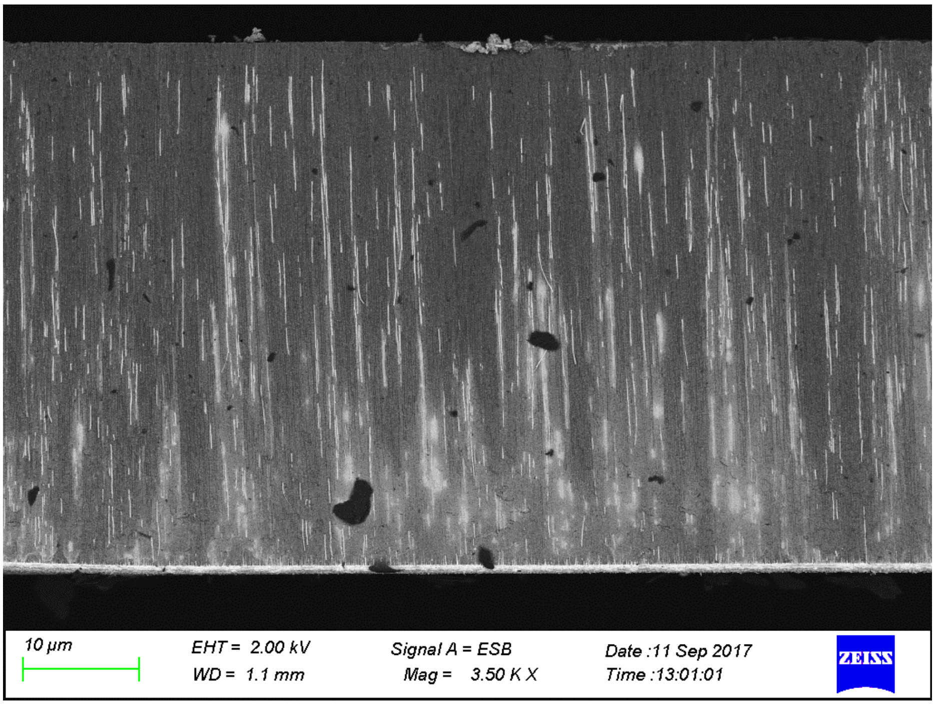

Fig. S11 Cross-sectional SEM image of an anodic alumina membrane with 55 nm diameter pores that have been grafted with $C_2H_5Si(OCH_3)_3$. Tin nanowires through the pores can be seen in white.
